# Does Stage at Diagnosis Affect Prognosis of Patients With Stage IV Breast, Lung, and Colorectal Cancers?

**DOI:** 10.1093/jncics/pky025

**Published:** 2018-06-28

**Authors:** Belinda E Kiely

**Affiliations:** NHMRC Clinical Trials Centre, University of Sydney, Sydney, NSW, Australia

Estimating survival time for patients with advanced cancer is difficult, and data that help oncologists more accurately estimate survival time are important. Survival estimates from clinical trials provide a good starting point, but in routine clinical practice, trial estimates need to be adjusted to account for differences in patient performance status, extent of disease, comorbidities, and response to treatment. Another factor to consider is whether a patient has relapsed after treatment for early-stage cancer or presented with de novo metastatic disease.

The accompanying article by Hassett et al. (1) compares the survival time from diagnosis of advanced cancer of 733 patients with recurrent stage IV cancer (initially diagnosed with stage I–III cancer, completed local therapy then relapsed) with 733 patients with de novo stage IV cancer (presenting with stage IV cancer at diagnosis). There are three subsets: 219 matched pairs of patients (recurrent and de novo) with stage IV breast cancer (BC), 182 matched pairs with stage IV lung cancer (LC), and 332 matched pairs with stage IV colorectal cancer (CRC). The key findings are a 6.8-month longer average survival for de novo vs recurrent BC (29 vs 22.2 months), a 3.4-month shorter average survival for de novo vs recurrent LC (18.9 vs 22.3 months), and no significant survival difference for de novo vs recurrent CRC (23.8 vs 25.4 months). More de novo vs recurrent patients received chemotherapy for stage IV disease (BC: 85% vs 65%; LC: 86% vs 56%; and CRC: 78% vs 64%). When patients with regional recurrences only (and no distant metastases) were excluded, survival remained longer for de novo vs recurrent BC but was equal for de novo vs recurrent LC and CRC. The longer survival of recurrent LC patients was seemingly due to regional recurrences.

So why might we see a difference in survival between de novo and recurrent stage IV cancers? First, we need to consider differences in how patients are diagnosed. Patients with de novo metastatic cancer frequently present with symptoms from their metastases. Such patients have a high disease burden and typically start systemic therapy to rapidly gain control of their disease and reduce symptoms. In contrast, recurrent metastatic patients are often diagnosed during post-treatment surveillance when they are asymptomatic with low-volume disease. Following a diagnosis of early-stage CRC regular serum carcinoembryonic antigen levels, computed tomography (CT) scans and colonoscopies are standard care because of a proven survival benefit (2,3), and many patients undergo resection of limited metastatic disease. Although there is less evidence for surveillance in early-stage LC (4), patients often undergo regular chest x-rays and CT scans following definitive therapy. Once diagnosed, asymptomatic recurrent patients may prefer to “watch and wait,” delaying chemotherapy until symptomatic. Patients with recurrent stage IV CRC and LC are therefore more likely to be diagnosed with low-volume disease and, due to lead time bias, could be expected to experience longer survival than the matched de novo patients. This is not the case in early BC, where guidelines recommend against post-treatment surveillance because there is no proven survival benefit (5,6). As a result, both recurrent and de novo stage IV BC patients tend to be diagnosed when symptomatic, but their survival differs.

Survival was shorter for the recurrent BC patients, most of whom (72%) had received adjuvant chemotherapy. Recurrence after adjuvant chemotherapy is a marker of treatment resistance, so intuitively we would expect these patients to have shorter survival. However, a similar proportion of recurrent CRC patients had received adjuvant chemotherapy (70%), yet their survival was similar to patients with de novo CRC. This could be partly explained by earlier detection of recurrent CRC compared with recurrent BC, but also the increased number of effective treatments for stage IV CRC (eg, irinotecan, cetuximab, and bevacizumab), compared with the limited effective adjuvant therapies for CRC. Treatment resistance may be more pronounced in stage IV BC because similar chemotherapies and targeted therapies are used in both the adjuvant and metastatic settings. Adjuvant chemotherapy rates were much lower (40%) in recurrent LC, so treatment resistance was less of a concern, and again there are more treatment options in the metastatic setting.

The matching algorithm ensured that patient demographics were well balanced between recurrent and de novo groups; however, patient performance status, sites and burden of metastatic disease, tumor molecular subtypes, and receipt and type of systemic therapy were not matching criteria. Given the relatively small sample size, it is possible that differences in the distribution of these important prognostic factors may have impacted the observed survival times. With targeted therapies such as trastuzumab, gefitinib, and cetuximab, survival of some patients with metastatic cancer can be significantly prolonged, and any imbalance in molecular subtype between recurrent and de novo patients could explain the reported differences in survival. For example, den Brok et al. found that the median survival for patients with metastatic BC varied significantly by subtype as well as by de novo vs recurrent diagnosis: estrogen receptor–positive (ER+) 34 vs 23 months, human epidermal growth factor receptor 2–positive 29 vs 15 months, and triple-negative 11 vs 8 months. (7).

Disease-free interval (DFI) for recurrent stage IV patients also needs to be considered. Longer DFI is a known positive prognostic factor and is suggestive of more indolent underlying disease biology, especially in recurrent BC where late recurrences are common in ER+ disease. Of note, two publications reporting longer survival for de novo vs recurrent metastatic BC found that the survival difference was only evident when the DFI was less than five years (7,8). When the DFI was more than five years, patients with recurrent metastatic BC had similar survival to those with de novo disease (7,8).

The findings of Hassett et al. are interesting and hypothesis generating. The significantly longer survival seen in de novo vs recurrent metastatic BC in this and other studies supports oncologists accounting for stage at diagnosis when estimating survival for their patients with advanced BC. Given that estimates of survival time help patients make treatment decisions and plans for the future, improving the accuracy of survival estimates is very important. Stratification of patients with metastatic BC in phase III clinical trials based on stage at diagnosis should also be considered. Further exploration in larger data sets matched for molecular subtype and extent of metastases is required in LC and CRC before a similar recommendation can be made.

## Funding

Funding for research from an NHMRC Clinical Trials Centre program grant

## Note

Affiliation of author: NHMRC Clinical Trials Centre, University of Sydney, Sydney, NSW, Australia.

Honoraria and conference support from Roche, Novartis and Teva.
